# l-Dopa and Brain Serotonin System Dysfunction

**DOI:** 10.3390/toxics3010075

**Published:** 2015-03-05

**Authors:** Branden J. Stansley, Bryan K. Yamamoto

**Affiliations:** Department of Neurosciences, University of Toledo College of Medicine, 3000 Arlington Ave, Toledo, OH 43561, USA; E-Mail: branden.stansley@utoledo.edu

**Keywords:** 6-hydroxydopamine, dopamine, l-dopa, non-motor symptoms, Parkinson’s disease, serotonin neurons

## Abstract

l-dopa is used to treat the motor symptoms associated with Parkinson’s disease, a neurodegenerative movement disorder characterized by a loss of dopamine neurons. l-dopa is the precursor to dopamine and crosses the blood-brain barrier to increase dopamine neurotransmission. This review will focus on the findings that dopamine produced from l-dopa is mediated in part by serotonin neurons. Direct evidence will be provided that increases in dopamine cause oxidative stress and damage serotonin neurons. Similarly, chronic l-dopa produces deficits in serotonin neurotransmission, including decreases in both serotonin cell bodies within the dorsal raphe and serotonin neurotransmitter concentrations in several forebrain regions. Since serotonin is involved in many important physiological processes including mood and cognition, l-dopa induced serotonin deficits may play a role in the side-effect symptoms observed in Parkinson’s disease patients treated with l-dopa.

## 1. Introduction

Parkinson’s disease (PD) is the second most common neurodegenerative disorder behind Alzheimer’s, affecting nearly a million people in the United States [1]. The disease is characterized by a degeneration of dopamine neurons in the substantia nigra [2] that project axons to the striatum, release dopamine and thus influence motor behavior [3]. The resulting dopamine deficits produce the classic motor symptoms associated with PD, such as bradykinesia, akinesia, and tremor [4]. Several pharmacological therapies are effective at alleviating these motor symptoms including dopamine agonists and monoamine-oxidase (MAO) inhibitors [5]. However, l-dopa has proven to be the most reliable and efficacious of the current PD therapeutics for ameliorating motor deficits in patients with PD. The discovery of l-dopa’s ability to abolish reserpine induced motor symptoms in mice [6] was followed closely by the clinical use of l-dopa for the treatment of Parkinsonism in humans [7]. To date, l-dopa remains the benchmark therapeutic for the restoration of dopamine in PD.

l-dopa is not without its negative side-effects however, as increased treatment duration is associated with the occurrence of dyskinesia [8] and hallucinations [9]. Furthermore, many non-motor symptoms are associated with PD itself, such as depression, anxiety, sleep disorders, and cognitive impairments [10]; all of which greatly impact the overall quality of life of the patient. Interestingly, some clinical reports suggest that these symptoms are not improved and may be exacerbated by l-dopa treatment [11,12,13]. Both the negative side-effects of l-dopa, as well as the non-motor symptoms of PD may involve the brain serotonin (5-HT) system. Further, l-dopa exerts direct effects on the 5-HT systems because 5-HT neurons are capable of taking up l-dopa and decarboxylating it to dopamine [14,15], eventually releasing dopamine in an impulse dependent manner [16]. Moreover, the accumulation of dopamine to supraphysiologic concentrations has been shown to be toxic to 5-HT neurons through oxidative mechanisms [14,17]. This review will summarize the findings that l-dopa can be toxic to central 5-HT neurons and result in 5-HT related deficits that could lead to overall physiological dysfunction in PD patients after chronic l-dopa therapy.

## 2. l-Dopa Treatment in PD

l-dopa is often administered orally several times a day to PD patients due to its relatively short plasma half-life (~60–90 min) [18]. l-dopa is often combined with a peripheral decarboxylase inhibitor, such as carbidopa, to prevent its conversion to dopamine in the periphery and allow for greater concentrations of l-dopa to reach the central nervous system (CNS) [19]. Unlike dopamine, l-dopa is transported across the blood-brain barrier via the l-system large neutral amino acid transporter [20]. This ubiquitous transport system is found throughout the blood-brain barrier [21] and has high affinity for neutral amino acids. The large systemic dosing of l-dopa results in the production of supraphysiologic dopamine concentrations throughout the brain. While the therapeutic dose of l-dopa partially restores extracellular dopamine concentrations in the denervated striatum, other areas that normally contain little or no dopamine also have significantly increased dopamine concentrations [22]. This is explained by the fact that after transport into the CNS, l-dopa is then converted into dopamine by aromatic amino-acid decarboxylase (AADC), an enzyme that is not specific to dopamine neurons but is found in many different cells including glia and endothelia, as well as 5-HTergic neurons [23,24,25,26]. In fact, 5-HT neurons have been shown to be responsible for the majority of l-dopa induced dopamine release within the striatum of 6-hydroxydopamine (6-OHDA) lesioned rats as well as extrastriatal brain regions such as the substantia nigra pars reticulata, hippocampus and prefrontal cortex [22,27,28,29,30].

### 2.1. l-Dopa and the 5-HT System

5-HT neuron soma from the midbrain send projections throughout the entire CNS. This important neurochemical system is one of the most evolutionarily preserved and prevalent in the mammalian brain. It has been demonstrated that one 5-HT cell body can be responsible for up to 500,000 cortical varicosities [31], highlighting the dense network of innervations derived from the 5-HT neurons clustered within the midbrain raphe nuclei (areas B_1_-B_9_) [32]. These nuclei include the dorsal, median, magnus, obscures, and pontis raphe regions [33]. The 5-HT projections to the forebrain regions are derived primarily from the doral raphe (DRN) and median raphe nuclei (MRN) [34,35]. Within the DRN, the 5-HT neurons are topographically organized further into sub-regions to include the (1) rostral; (2) ventral-medial; (3) dorsal-medial; (4) interfascicular; (5) lateral-wings; and the (6) caudal DRN. Studies have also shown that 5-HT neurons within each DRN sub-region differ morphologically, electrophysiologically, and molecularly with regard to receptor expression [36]. Additionally, DRN sub-regions all possess different afferent and efferent projections [37]. These projections to and from the DRN are important with regard to PD treatment, disease progression and symptomatology. For instance, the ventral-medial DRN sub-region projects densely to the basal ganglia structures and motor cortices [37], while the dorsal-medial DRN sends axons to limbic areas such as the amygdala, nucleus accumbens, and medial-prefrontal cortex [38]. However, as previously mentioned, all 5-HT neurons are similar in that they possess the ability to uptake l-dopa [39], decarboxylate it to dopamine [40], and package the dopamine into vesicles for exocytotic release [41].

It was previously assumed that l-dopa is decarboxylated into dopamine within the spared nigrostriatal dopaminergic neurons of Parkinson’s disease patients or 6-OHDA/1-methyl-1,4-phenyl-1,2,3,6-tetrohydropyridine (MPTP) rodent models, and that the dopamine released from these dopaminergic terminals within the striatum accounts for the therapeutic benefits of l-dopa. However, several studies have provided evidence for 5-HT neurons as the main means by which dopamine is synthesized from exogenous l-dopa and released in the striatum, thus accounting for l-dopa’s therapeutic effects on movement in PD. An additional consequence of systemic l-dopa administration is that dopamine concentrations are elevated in “off-target” brain areas in a pattern that follows 5-HT innervations [22]. In fact, areas that are mostly devoid of dopaminergic innervations but possess a significant degree of 5-HT innervation have increased dopamine after l-dopa. This is exemplified by the fact that unilateral 6-OHDA lesioned rats have a greater increase in striatal extracellular dopamine levels ipsilateral to the dopaminergic lesion compared to the intact side, suggesting that non-dopaminergic and perhaps 5-HTergic innervations are responsible for the increased dopamine release [42]. Indeed, the striatum receives dense 5-HT innervation from the dorsal raphe [37], and is actually hyperinnervated by 5-HT axons after 6-OHDA lesioning in rats [43]. This phenomenon by which dopamine is elevated within notably 5-HTergic brain regions after l-dopa has been investigated in numerous studies and validates the ability of 5-HTergic neurons to synthesize and release dopamine.

### 2.2. l-Dopa Induced Dopamine Production within 5-HT Neurons

Early studies utilizing striatal brain slices *in vitro* demonstrated that l-dopa induced production of dopamine occurs within 5-HT neurons and is dependent on AADC activity [15]. Furthermore, dopamine can be visualized immunohistochemically within 5-HT fibers of the striatum and the substantia nigra pars reticulata after systemic l-dopa administration to rats [24,44]. In addition, work from our laboratory has shown that within the RN46A-B14 cell line, a homogenous 5-HTergic cell line derived from the raphe, exogenous l-dopa is decarboxylated to dopamine in a manner that is blocked by the AADC inhibitor NSD-1015 [14].

Neuroanatomical approaches also have been used to examine the contribution of 5-HT neurons toward increased tissue and extracellular concentrations of dopamine after l-dopa. This is highlighted by a study using 6-OHDA treated rats in which 30 mg/kg l-dopa failed to increase dopamine content in the striatum with lesions of the raphe produced by the 5-HT neurotoxin 5,7-dihydroxytriptamine (5,7-DHT) [28]. This study provides further supportive evidence that 5-HT axons contribute to l-dopa-induced dopamine in the striatum after nigrostriatal dopaminergic degeneration. These findings were expanded upon by a more recent study that used *in vivo* microdialysis to measure increases in extracellular dopamine after l-dopa in the lesioned striatum, as well as in the intact substantia nigra pars reticulata, hippocampus, and prefrontal cortex. The results indicated that l-dopa dose dependently increased extracellular dopamine in all brain regions, and that these effects were abolished when raphe 5-HT neurons were destroyed by 5,7-DHT [22].

Moreover, l-dopa induced increases in dopamine can be controlled when 5-HT neurons are pharmacologically manipulated. l-dopa-induced increases in extracellular dopamine in the striatum and substantia nigra are prevented by the administration of 5-HT_1A/1B_ agonists, suggesting that inhibition of 5-HT neuron firing by activation of 5-HT autoreceptors, prevents the exocytotic release of dopamine from 5-HT nerve terminals [30,45]. Overall, multiple studies show the off-target actions of exogenous l-dopa in synthesizing supraphysiologic concentrations of dopamine within 5-HT neurons.

### 2.3. 5-HT Neurons and l-Dopa Induced Dyskinesia

l-dopa induced dopamine within 5-HT neurons also contributes to negative motor side-effects in PD patients. Clinically, the benefit of motor symptom relief provided by l-dopa to PD patients is often usurped by severe dyskinesia or uncontrollable involuntary movements that develop after chronic treatment [46]. Dyskinesias are highly prevalent and have been associated with decreased quality of life in PD patients [47]. Until recently, the involvement of 5-HT neurons in l-dopa-induced dyskinesia was underappreciated; however, several studies have provided convincing evidence in animal models [48,49] and human PD patients [50] that validate the contribution of 5HT neurons to l-dopa-induced dyskinesias. A study by Carta *et al.* showed that aberrant dopamine release by 5-HT innervations of the striatum is responsible for dyskinesia and that removal of 5-HT afferents by a lesion of the DRN, or agonism of 5-HT1A/1B receptors blocked l-dopa induced dyskinesias in 6-OHDA rats [49]. Furthermore, others have shown that chronic l-dopa treatment can result in a maladaptive plasticity of 5-HT neuron fibers projecting to the striatum in animals that have both severe dopaminergic degeneration, as well as partial 5-HT axon lesions, leading to greater striatal dopamine release and dyskinesias [51]. These findings were corroborated by human studies showing that [^11^C]-DASB binding, a marker of SERT, was greater in dyskinetic PD patients compared to stable responders, suggestive of an increase in striatal 5-HT terminals [50]. Therefore, chronic l-dopa treatment appears to cause 5-HT terminal sprouting in the striatum that results in dysregulated l-dopa-induced dopamine release and dykinesias in PD.

## 3. l-Dopa Induced 5-HTergic Deficits

Another consequence of this “off-target” effect is that l-dopa can become toxic to 5-HT systems. One mechanism of 5-HT neurotoxicity appears to be related to oxidative stress produced by l-dopa-induced supraphysiologic concentrations of dopamine. Dopamine has long been known to be a potent oxidant [52], as unsequestered dopamine can serve as a pro-oxidant when it auto-oxidizes into quinone species. Alternatively, oxidative stress can be produced when dopamine is metabolized by the enzyme MAO to form the dopamine metabolite 3,4-dihydroxyphenylacetic acid and the by-product hydrogen peroxide [53]. This dopamine dependent oxidative stress has been shown to damage vital cell organelles such as mitochondria [53] and catecholaminergic cells by dopamine-quinone production [54] and dopamine metabolism by monoamine oxidase [55]. Similarly, dopamine has been shown to damage cellular proteins in 5-HTergic neurons. Tryptophan hydroxylase (TPH), the rate limiting enzyme in 5-HT production, is inactivated by dopamine-quinones [54]. Additionally, *in vitro* studies have demonstrated that dopamine can produce cell death in 5-HTergic cell culture via reactive oxygen species production resulting from the synthesis and degradation of dopamine after exogenously applied l-dopa [14].

l-dopa induced 5-HT cell toxicity has been demonstrated *in vivo* as well. In a unilateral 6-OHDA rat model, chronic l-dopa (6 mg/kg; 12 mg/kg benserizide; twice daily) administration for 10 consecutive days significantly decreased 5-HT cell bodies (cells co-labeled for TPH+/NeuN+) in the DRN, whereas 6-OHDA lesion had no effect [17]. Furthermore, the loss of 5-HT cell bodies was specific to the caudal extent of the dorsal sub-region of the DRN, an area that was found to have higher dopamine turnover after acute l-dopa compared to the ventral sub-region. The MAO type B inhibitor deprenyl, as well as ascorbic acid pretreatment prevented the l-dopa induced loss of 5-HT neurons within the DRN, demonstrating that dopamine degradation and oxidative stress contribute to the l-dopa induced damage to 5-HT systems [17]. These data are consistent with previous work demonstrating that TPH2 expression in the DRN is decreased in rats with bilateral 6-OHDA lesions and exacerbated by chronic l-dopa treatment [56], but are somewhat at variance with human post-mortem studies that did not find differences in DRN TPH+ neuron number when comparing dyskinetic to non-dyskinetic PD patients [57]. That study, however, was limited to l-dopa treated PD patients with or without dyskinesias such that there may have been a pre-existing 5-HT neuron loss in both groups due to the history of exposure to l-dopa. Furthermore, although neurons in the DRN as a whole have been shown not to be affected [58], 5-HT neurons in the caudal DRN were particularly susceptible to l-dopa induced damage [17]. These differences could be related to the fact that the dorsal sub-region of the DRN has a higher dopamine turnover rate after acute l-dopa than the ventral DRN, leading to greater oxidative stress after dopamine degradation by MAO. Furthermore, the lack of l-dopa induced damage to nigral dopaminergic neurons [59,60] could be explained by autoregulatory mechanisms, such as D2 receptors, which have been shown to decrease AADC activity and thus decrease conversion of l-dopa to dopamine when activated [61]. To our knowledge, 5-HT neurons lack any autoregulatory mechanisms for the production of dopamine and thus exacerbates dopamine production in 5-HT compared to dopaminergic neurons.

In addition to l-dopa damage to 5-HT neurons at the cell body level, l-dopa also exerts effects on 5-HT neurotransmitter production and release at the level of 5-HT axon terminal throughout the brain. Several reports indicate that both acute, as well as chronic, l-dopa can result in 5-HT deficits. Acutely, l-dopa maximally but transiently increases dopamine concentrations in the rodent brain within 30 min [62]. The increase in dopamine results in a consequent decrease in 5-HT tissue concentrations in both DRN and projection regions, which appear to fully recover within three hours [22]. The acute l-dopa induced decrease in 5-HT content is thought to be caused by high amounts of l-dopa, which compete with tryptophan for uptake by the amino-acid transporter on 5-HT neurons and subsequently for AADC. In addition, dopamine derived from l-dopa can displace existing 5-HT from vesicles [49,63,64]. Chronic l-dopa has also been shown to decrease 5-HT tissue content, even when l-dopa is no longer present. Rats treated chronically with high dose l-dopa (250 mg/kg/day) have depleted 5-HT tissue content throughout brain when measured 24 h after last l-dopa dose [63]. Furthermore, decreases in 5-HT and 5-HIAA tissue content were seen in the striatum and motor cortex after treatment with a more moderate dose (12 mg/kg/day for 10 days) [65]. Similar decreases in 5-HT content were observed within the DRN and prefrontal cortex of rats treated with 6 mg/kg of l-dopa twice daily for 10 days [17].

In addition to decreases in 5-HT tissue content within several brain areas, extracellular 5-HT was also decreased after chronic l-dopa. Navailles *et al.* have shown that basal extracellular 5-HT was lower in chronic l-dopa treated rats compared to controls. Specifically, chronic l-dopa caused a decrease in extracellular 5-HT and 5-HIAA in the striatum, substantia nigra pars reticulata, hippocampus, and prefrontal cortex. Furthermore, they also found that acute l-dopa treatment resulted in significantly less dopamine release in these regions, suggesting that these 5-HT terminals that normally release dopamine after l-dopa are compromised [65]. Overall, there is substantial pre-clinical evidence for the negative impact of chronic l-dopa on the 5-HT system. Some of these findings are summarized in Table 1.

**Table 1:** A selection of studies highlighting the 5-HT deficits caused by chronic l-dopa.

Model	l-Dopa concentration (mg/kg/day)	Treatment duration (days)	5-HTerigc deficit(s)	Brain region(s) affected	Reference
Rat (non-lesioned)	250	60	↓ 5-HT tissue content	STR, Cortex	Borah and Mohanakumar [63]
Rat (unilateral 6-OHDA)	12	10	↓ 5-HT tissue content	STR, Cortex STR, HIPP, SNr, PFC	Navailles *et al.* [65]
			↓ 5-HT extracellular		
Rat (unilateral 6-OHDA)	12	28	↓ 5-HT tissue content	Amygdala	Eskow-Jaunarajs *et al.* [66]
Rat (bilateral 6-OHDA)	12	75	↓ 5-HT tissue content	STR, Amygdala, PFC	Eskow-Jaunarajs *et al.* [58]
Rat (non-lesioned)	12	10	↓ 5-HT cell bodies	Dorsal DRN	Stansley and Yamamoto [17]
			↓ 5-HT tissue content	Dorsal DRN, PFC	
Macaque (MPTP-lesioned)	40	~90 (3 months)	↓ 5-HT tissue content	STR, Motor cortex, HIPP, Amygdala	Engeln *et al.* [67]

Chronic treatment with l-dopa to PD patients often results in a loss of drug efficacy [68], possibly because of damage to the 5-HT system that is dependent on l-dopa induced dopamine production and release [65]. Further clinical studies are needed to confirm these pre-clinical results suggesting that l-dopa may be detrimental to central 5-HT systems. In this regard, one study found a depletion of 5-HT concentration within the cerebrospinal fluid in l-dopa treated PD patients compared to untreated PD controls [69]. However, interpretation of such studies is somewhat confounded by the fact that PD itself results in a degeneration of 5-HT cell bodies in the DRN, as well as 5-HT markers throughout the brain [34]. Regardless, there has been a growing appreciation for 5-HT system involvement in PD therapeutic treatments, which will hopefully lead to more clinical research on l-dopa induced alterations of the 5-HT system.

## 4. Behavioral Implications of l-Dopa Induced 5-HTergic Deficits

Non-motor symptoms of PD are gaining attention by researchers and physicians [70], and perturbations in the 5-HT system are thought to contribute largely to these symptoms [71]. Under physiologic conditions, the brain 5-HT system maintains a strong influence over many important behavioral processes including but not limited to sleep, motor behavior, cognition, and emotion [72,73]. Alterations in the 5-HT system have been implicated in many pathological brain disorders, such as depression, anxiety, and schizophrenia [38,74,75]. Many such 5-HT associated impairments have been noted in PD patients including autonomic dysfunction, sleep disturbances, depression, and anxiety [10,76]. In fact, one study found that depression ranks as the most important factor in PD patients quality of life, even above disease severity [77]. Indeed, the non-motor symptoms are very prevalent in PD, with one study citing a nearly two-fold higher rate of depression within PD patients compared to those without PD [78]. Interestingly, PD progression results in degeneration of the DRN 5-HT neurons which precedes the loss of nigral dopamine neurons, although not to the same extent [79]. In fact, PD patients with depression have been shown to have more severe DRN cell loss compared to PD patients without depression [80]. Therefore, it is conceivable that l-dopa induced 5-HT deficits may further exacerbate existing behavioral impairments in PD patients. In support of this concept, affective disorders are not improved by l-dopa treatment [12,81], and may in fact can be worsened by chronic l-dopa therapy [11,13].

Deficits in 5-HT caused by acute and chronic l-dopa administration may dysregulate crucial brain areas such as the striatum and the frontal cortex that control motor function and cognitive stability. Both brain regions depend on cross-talk between 5-HT and dopamine to regulate behavior [82,83,84,85]. For example, many negative symptoms that appear during “ON” periods of l-dopa, such as dyskinesia and hallucinations, are thought to result from the imbalance of dopamine and 5-HT within the striatum and frontal cortex, respectively [86,87,88]. This overall dysregulation and imbalance of dopamine and 5-HT is also apparent pre-clinically in bilateral 6-OHDA rats as well as MPTP-lesioned macaques that were chronically treated with l-dopa [58,67]. As previously discussed, preclinical evidence for l-dopa induced deficits in 5-HT have been demonstrated in areas critical for cognitive and affective behaviors such as the amygdala, striatum, hippocampus, DRN, and prefrontal cortex [17,49,58]. Deficits in these brain regions could impact affective and cognitive behaviors such as depression, anxiety, fear-response and spatial memory [89,90,91,92]. This is supported by the finding that 12 mg/kg of l-dopa administered for 75 days produced anxiety like behaviors in bilaterally lesioned 6-OHDA rats, however it did not exacerbate negative indices such as depressive behavior [58]. Other rats treated with high doses of l-dopa for 60 days produced 5-HT deficits in several brain regions and also produced greater immobility in the forced swim task, suggesting chronic l-dopa induces depression-like behavior [63]. Alterations in 5-HT and affective behaviors are also observed in the rat PD model. This point is exemplified by a recent study by Santiago *et al.* that demonstrated depressive-like behaviors accompanied by reductions in hippocampal 5-HT after 6-OHDA lesioning in rats [93]. In contrast, another study found l-dopa actually produced an antidepressant effect in 6-OHDA rats, although treatment duration was only for three days and behavioral assays were conducted during the l-dopa ON phase [94]. Collectively, these studies signify the necessity for further research on the non-motor symptoms that could be attributed to 5-HT related deficits in PD. A mechanistic connection between the l-dopa induced 5-HT deficits and behavior could help to improve PD therapy, possibly by minimizing the non-motor impairments produced by l-dopa.

## 5. Conclusions

Current research indicates that dopamine produced by chronic l-dopa at therapeutically relevant doses has detrimental effects on 5-HT systems, which may lead to cognitive impairments and ultimately impact quality of life of the PD patient. While current data support the role of l-dopa for inducing 5-HTergic deficits, more research is needed to further elucidate the degree to which 5-HT brain physiology is altered, and to what extent these 5-HT deficits contribute to non-motor symptoms in PD. Future work aimed at elucidating mechanisms by which l-dopa acts on 5-HT neurons will allow for the development of therapeutics that relieve both the motor and non-motor symptoms associated with PD. Although l-dopa is the most effective treatment for the motor symptoms of PD, the elucidation of the mechanisms that underlie the off-target and toxic side-effects to the 5-HT system and non-motor behaviors may help improve l-dopa therapy and warrants further investigation.
